# Acute Hyperglycemia Due to Topical Corticosteroid Administration

**DOI:** 10.1155/2019/6058076

**Published:** 2019-01-02

**Authors:** Laura Y. Sue, Anna Milanesi

**Affiliations:** ^1^VA Greater Los Angeles Healthcare System and Division of Endocrinology, Diabetes and Hypertension, Department of Medicine, UCLA David Geffen School of Medicine, 900 Veteran Avenue, 24-130 Warren Hall, Los Angeles, CA 90095, USA; ^2^Department of Medicine, Veterans Affairs Greater Los Angeles Healthcare System and Division of Endocrinology, Departments of Medicine and Physiology, UCLA David Geffen School of Medicine, 11301 Wilshire Boulevard, Los Angeles, CA 90073, USA

## Abstract

We present the case of a 71-year-old man with longstanding, previously well-controlled type 1 diabetes who developed acute hyperglycemia. His insulin requirements, via his insulin pump, increased to nearly five times his typical daily dose. The patient was admitted for evaluation and treatment and started on an insulin infusion. He had minimal insulin requirements with the insulin infusion. History revealed recent use of a super potent topical corticosteroid for a psoriasis flare. The patient was transitioned back to his insulin pump, using his prior to admission settings. He was advised to discontinue using his topical corticosteroid. He had no further hyperglycemic episodes. The clinical presentation is suggestive of corticosteroid-induced hyperglycemia, suggesting that clinically significant changes can occur even with short duration use, particularly with high potency steroids used. This is to our knowledge the first case reported in which the patient required a very significant amount of extra insulin (nearly five times his typical total daily dose) after using high potency topical steroid cream. This case highlights the potentially detrimental effect of topical corticosteroid use in patients with diabetes.

## 1. Introduction

Systemic/oral administration of corticosteroids can induce or worsen hyperglycemia; however, there are limited reports about topical formulations of corticosteroids causing hyperglycemia. Here we report a case of acute hyperglycemia related to topical corticosteroid use in a patient with longstanding, well-controlled diabetes.

## 2. Case Presentation

We cared for a 71-year-old man with a 45-year history of type 1 diabetes. He had used an insulin pump for years with a current daily dose of 73 units of insulin. His hemoglobin A1C values had ranged from 5.9 to 7.4% in the last ten years.

**Figure 1:**
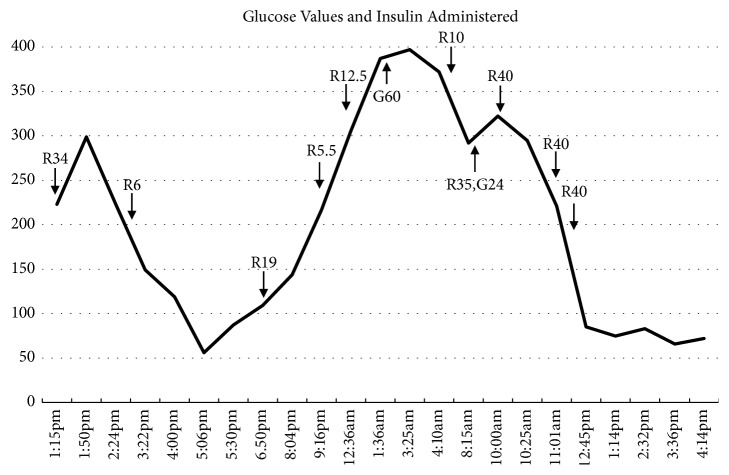
Glucose values (solid line) and insulin administered by the patient (R: regular insulin; G: glargine insulin; number denotes the number of units given) over time on the day prior to and the day of presentation.

The day prior to admission, the patient developed sudden persistent hyperglycemia. He required 326 units of insulin injections within 24 hours, in addition to the 30 units of basal insulin via his pump. His glucose finally decreased to 85 mg/dl six hours before presentation to the emergency department (see Figure 1). He had normal vitals on presentation. Evaluation, including complete blood count, chemistry panel, blood cultures, and chest radiograph, was normal. Cortisol was not measured.

Upon obtaining further history, the patient reported being in his usual state of health except for a psoriasis flare for which he had used fluocinonide 0.1% cream in the two days prior to presentation. Although he had used a small amount of cream on his hands before, this time he applied the cream to a larger area, including his abdomen, twice a day, using occlusive techniques to increase effectiveness. While hospitalized, the patient was kept on an insulin infusion, requiring 0.25-3 units/hour. On hospital day 2, he was transitioned to his insulin pump using his prior-to-admission settings. He was discharged home and advised to discontinue using the fluocinonide cream. On outpatient follow-up, he did not report further hyperglycemia.

## 3. Discussion

The clinical presentation is suggestive of corticosteroid-induced hyperglycemia. We know that systemic/oral administration of corticosteroids can induce or worsen hyperglycemia; however, there are limited reports about topical use causing hyperglycemia. Topical corticosteroids are classified by potency from class I (super potent) to class VII (least potent) [1]. The fluocinonide 0.1% cream that our patient used is in class I. It has been hypothesized that topical corticosteroids cause hyperglycemia through increasing alanine transport, thereby facilitating hepatic gluconeogenesis, increasing enzymatic activity, and causing a relative insulin resistance [2].

Gomez and Frost reported two patients with abnormal glucose tolerance tests who developed postprandial hyperglycemia with topical administration of high potency corticosteroid creams [3]. Two other patients, despite also having abnormal pretreatment glucose tolerance tests, had no changes in postprandial glucose values. All patients had decreased levels of measured cortisol, suggesting clinically significant systemic effects of the topical corticosteroids. Hongo et al. described an elderly man with longstanding diabetes who was diagnosed with psoriasis [1] and switched from oral to topical corticosteroid therapy. His glycemic control worsened and he required a 76% increase in total daily insulin dose (Table 1). Kahara et al. reported the case of a patient treated for oral lichen planus with a class VII (lowest potency) steroid on his oral mucosa. The patient developed new onset diabetes that required initiation of an oral anti-diabetes medication for several months (Table 1) [4]. A nested case-control study of over 192,000 patients in the Netherlands found a 1.24-fold increased risk of developing diabetes among those currently using topical corticosteroids. Risk was even higher among patients with more than 180 days of topical corticosteroid use [odds ratio (OR) 1.32, 95% confidence interval 1.14-1.54)] and among those with higher cumulative exposure (ORs ranging from 1.29 to 1.44; statistically significant) [5]. In addition to hyperglycemia, there have also been reported cases of Cushing syndrome and adrenal crisis due to topical corticosteroid use [2].

Although there are limited data about topical corticosteroid therapy causing alterations in glucose metabolism, it appears that clinically significant changes can occur even with short duration use, particularly with high potency steroids used with occlusive techniques for increased effect. This is to our knowledge the first case reported in which the patient required a very significant amount of extra insulin (nearly five times his typical total daily dose) after using high potency topical steroid cream. Hence, clinicians need to be aware of the potentially detrimental effect of topical corticosteroid use in patients with diabetes.

## Figures and Tables

**Table 1:** Comparison of the current case with other cases of hyperglycemia related to topical corticosteroid use.

	**Patient Information**	**Duration of Diabetes**	**Details of Topical Corticosteroid Use**	**Glycemic Control**	**Diabetes Regimen Prior**	**Diabetes Medications During Hyperglycemic Period**
Current case	71 year old Caucasian man with type 1 diabetes	45 years	Psoriasis treated in the preceding 2 days with fluocinonide 0.1% cream (class I steroid)	A1C 5.9-7.4% in the prior 10 years	Insulin pump: 73 units total daily dose	Insulin via pump and subcutaneous injections: 356 units in 24 hours

Hongo et al 2017	80 year old Japanese man with type 2 diabetes	16 years	Psoriasis treated with class III-class V steroids over a 7-8 week period	First admission: A1C 8-9%; second admission 10 days later: A1C 7.6%	Lispro insulin 42 units per day, voglibose 0.9 mg per day	First admission: insulin increased to a maximum of 36 units of neutral protamine Hagedorn (NPH) insulin and 38 units of lispro insulin per day; second admission: insulin 32 units per day (dosing by insulin type not specified)

Kahara et al 2016	68 year old Japanese man with no history of diabetes	-----	Oral lichen planus treated with class VII steroid on the oral mucosa over a 6 month period	A1C 10.8% on admission after developing thirst and polyuria	-----	Alogliptin 25 mg daily started 2 months after diagnosis of diabetes and continued for 2 months
